# Increased visual reliance for postural control in multiple sclerosis: preliminary evidence from task-related cortical desynchronization

**DOI:** 10.3389/fneur.2026.1882165

**Published:** 2026-09-30

**Authors:** Leonard Braunsmann, Isabel Stolz, Franca Rosiny, Dieter Pöhlau, Vera Abeln, Stefan Schneider, Thomas Abel, Kristel Knaepen

**Affiliations:** 1 Institute of Movement and Neurosciences, German Sport University Cologne, Cologne, Germany; 2 Research Institute for Inclusion Through Physical Activity and Sports, Frechen, Germany; 3 DRK Kamillus Hospital, Asbach, Germany

**Keywords:** MS, balance, postural control, EEG, force plate, alpha, beta, sensory integration

## Abstract

**Background:**

Balance impairment is one of the most disabling symptoms in multiple sclerosis (MS), yet the underlying cortical mechanisms remain poorly understood. This exploratory study investigated how postural demands modulate brain activity in MS.

**Methods:**

Seven individuals with MS (mean age: 51.1 ± 9.7 years; 5 women; median Expanded Disability Status Scale: 5.5, IQR: 4.5–6.0) and seven age- and sex-matched healthy controls (mean age: 51.0 ± 9.6 years) performed three static balance tasks on a force plate: quiet stance with eyes open, quiet stance with eyes closed, and narrow stance with eyes open. Electroencephalography (EEG) and center of pressure (COP) were simultaneously recorded. Group differences were assessed using Linear Mixed Models. Significant effects are reported with false discovery rate (FDR)-corrected *q*-values, and otherwise with uncorrected *p*-values.

**Results:**

MS patients demonstrated greater postural sway than healthy controls, with FDR-corrected group × condition interactions across COP parameters (semi-partial *R*^2^ = 0.225–0.441; all *q* < 0.05), most pronounced during eyes closed (semi-partial *R*^2^ = 0.124–0.282; all *p* < 0.05). Resting-state EEG revealed no consistent group differences except for an uncorrected elevation of occipital theta power in MS patients (*p* = 0.017; *r* = −0.76). During stance, uncorrected group × condition interactions indicated that MS patients exhibited a greater task-induced decrease of theta, alpha, and beta power relative to their matched resting baseline compared to healthy controls (semi-partial *R*^2^ = 0.193–0.318; all *p* < 0.05). These effects were specific to the eyes-closed condition.

**Conclusion:**

These preliminary findings are consistent with an increased reliance on visual information for postural control in MS, accompanied by a task-related cortical desynchronization specifically upon visual deprivation. The observed effect sizes, while likely subject to inflation given the small sample size, suggest that oscillatory EEG parameters warrant investigation in larger confirmatory studies.

## 1 Introduction

Multiple sclerosis (MS) is a chronic inflammatory disease characterized by demyelination of the central nervous system (1). Among a range of motor dysfunctions, balance impairment represents one of the most disabling symptoms in MS (2, 3). These postural control deficits substantially reduce mobility and autonomy, increase fall risk and associated injuries, and negatively affect overall quality of life (4–6).

Posturography using force platforms is widely recognized as an objective and sensitive method to evaluate balance ability in individuals with MS by analyzing the center of pressure (COP) (7–9). During static balance tests, in which participants maintain a fixed posture, MS patients demonstrate an increased postural sway compared to healthy controls as evidenced by larger COP amplitudes, increased COP path lengths, and higher COP velocities (10, 11). These deficits are present even in individuals with minimal MS-related disability (2, 12–14). However, postural instability progressively worsens with increasing clinical impairment as measured by the Expanded Disability Status Scale (EDSS) (15, 16). Especially COP excursion in the medio-lateral direction has been identified as a critical predictor of fall risk (17). Postural deficits in MS become particularly pronounced under challenging sensory conditions, especially upon removal of visual input, as individuals with MS tend to rely more heavily on visual feedback for postural control compared to healthy individuals (18–20). These impairments suggest an underlying dysfunction in the dynamic integration of multiple sensory modalities required for postural stability (11).

To better understand the cortical mechanisms, electroencephalography (EEG) offers a non-invasive window into cortical contributions to postural control. Studies in healthy individuals provide an important reference framework, showing that postural demands modulate oscillatory activity across a distributed cortical network spanning fronto-central, centro-parietal, and occipital regions (21, 22). Higher postural demands are typically associated with reduced alpha power (8–13 Hz), especially in centro-parietal areas (23–25). This is thought to reflect increased cortical engagement for sensory information processing as alpha oscillations are linked to the active gating of task-irrelevant input (26, 27). Similarly, reduced beta power (13–30 Hz) was observed during walking on a balance beam compared to treadmill walking (28). Together, these task-related reductions in oscillatory power are referred to as task-related cortical desynchronization, reflecting increased cortical engagement during active task performance (29). Furthermore, increases in cortical theta activity (4–8 Hz) were observed particularly in fronto-central regions including the anterior cingulate cortex (24, 25, 28, 30). This theta modulation is associated with error detection and postural monitoring (25, 31). Notably, theta increases appear to be specific to mechanically destabilizing tasks. Studies challenging postural control through sensory manipulation on a stable surface have not consistently observed this pattern (23).

However, it is important to note that individuals with MS demonstrate altered brain activity with an overall inconsistent pattern (32). Resting-state EEG studies have reported heterogeneous abnormalities including shifts toward slower frequencies, reduced interhemispheric coherence, and altered alpha and delta activity (32–34). This suggests a generalized disruption of neural circuits, potentially involving both pathological slowing and compensatory reorganization of cortical networks (32). Beyond traditional frequency band analysis, the EEG power spectrum can be decomposed into periodic and aperiodic components, an approach that has only recently been applied to clinical populations (35). The aperiodic exponent reflects the slope of the 1/f power spectrum and is thought to index the balance between neural excitation and inhibition (E:I balance) (36), while the offset is associated with broadband cortical excitability, potentially reflecting overall neuronal spiking activity (37, 38). In MS, disruptions in E:I balance have been observed during resting state, with steeper spectral slopes associated with reduced cognitive performance (39) and greater MS-related disability (40). This might suggest that increased inhibition may reflect a compensatory mechanism attempting to maintain cortical function. Conversely, MS patients with higher cognitive fatigue exhibit flatter frontal aperiodic slopes (41), suggesting that E:I imbalance may vary depending on disease-specific factors. However, research on aperiodic parameters in MS remains scarce, and no studies have examined the aperiodic component or its modulation during postural tasks.

Furthermore, existing neurophysiological research in MS has focused primarily on dynamic balance conditions rather than static postural control. During gait individuals with MS exhibit increased beta (13–30 Hz) and gamma (31–50 Hz) power particularly in frontal and parietal areas (42). This has been interpreted as reflecting greater cognitive involvement for motor planning and sensory integration to compensate for impaired subcortical gait control. Consistent with this, increased prefrontal oxygenation during walking has been reported in MS despite comparable walking performance to healthy controls (43), suggesting that greater cortical resources are recruited to maintain functional motor output. Whether similar compensatory mechanisms extend to static postural control in MS has not yet been examined, representing a critical gap in our understanding of MS-related postural deficits. Thus, the present study compared oscillatory and aperiodic brain activity during static balance tasks of varying postural demands between individuals with MS and age- and sex-matched healthy controls. We hypothesize that individuals with MS exhibit greater postural sway than healthy controls, accompanied by reduced alpha and beta power and altered theta activity during stance. Based on the limited existing evidence on aperiodic EEG parameters in MS, we additionally explore whether postural demands modulate the aperiodic exponent and offset differently between groups.

## 2 Materials and methods

### 2.1 Study design

This investigation served as a preliminary study within the larger randomized controlled multicenter study called MS-HIPPO II (44). The study was conducted in accordance with the Declaration of Helsinki of 1964, and all procedures were approved by the ethic committee of the German Sport University Cologne (No. 163/2023) and the ethics commission Rhineland-Palatinate (No. 2023–17,325). Prior to data collection, a detailed verbal and written explanation of the study was provided, and all participants gave their written informed consent.

### 2.2 Participants

A total of 23 individuals were initially enrolled in the study. Nine participants were excluded due to poor EEG data quality resulting from excessive movement artifacts during the balance tasks, resulting in a final sample of 7 individuals with MS (mean age: 51.1 ± 9.7 years; 5 women; median EDSS: 5.5, IQR: 4.5–6.0) and 7 age- and sex-matched healthy controls (mean age: 51.0 ± 9.6 years). Demographic characteristics are presented in Table 1. Given the exploratory nature of this study, no formal power calculation was performed. Nevertheless, both posturography and EEG are recognized as sensitive methods for detecting balance-related group differences in MS (14, 32, 45), supporting the feasibility of detecting relevant effects even in small samples. The inclusion criteria for MS participants involved a previously neurologist-confirmed diagnosis of MS and an EDSS score between 4.0 and 6.5. To reduce selection bias, both inpatient (*n* = 4) and outpatient (*n* = 3) subjects were investigated. Inclusion criteria for healthy controls required no history of neurological or orthopedic pathology that might influence balance or mobility.

**Table 1:** Demographic and clinical characteristics.

Variable	MS (*n* = 7)	Healthy (*n* = 7)	Statistics
Age (years)	51.1 ± 9.7	51.0 ± 9.6	*t* = 0.03, *p* = 0.978
Female (*n*)	5	5	χ^2^ = 0.00, *p* = 1.000
Height (cm)	171.0 ± 6.5	171.9 ± 10.2	*t* = −0.19, *p* = 0.854
Weight (kg)	78.3 ± 19.4	71.6 ± 8.9	*t* = 0.84, *p* = 0.420
Body Mass Index (kg/m^2^)	26.7 ± 6.2	24.3 ± 3.0	*t* = 0.93, *p* = 0.369
EDSS (0–10)	5.5/IQR: 4.5–6.0	–	–

Values represent means ± SD, except Expanded Disability Status Scale (EDSS) which is reported as median/interquartile range (IQR). MS, participants with multiple sclerosis.

### 2.3 EEG and posturography recordings

On a single visit, we first conducted a seated resting-state EEG with eyes open (resting-EO) and eyes closed (resting-EC) for two minutes, respectively. We used 32 Ag/AgCl active electrodes (Fp1, Fp2, F7, F3, Fz, F4, F8, FT9, FC5, FC1, FC2, FC6, FT10, T7, C3, Cz, C4, T8, CP5, CP1, CP2, CP6, TP10, P7, P3, Pz, P4, P8, TP9, O1, Oz, O2) plus one reference electrode (FCz) and one ground electrode (Fpz) according to the international 10–20 system (46). Electrode impedance was kept below 10 kOhm using a SuperVisc electrogel (EASYCAP GmbH, Woerthsee-Etterschlag, Germany). The signal was amplified using LiveAmp (Brain Products GmbH, Gilching, Germany) with a sampling rate of 500 Hz. Subsequently, we assessed balance ability using a 60 (H) × 40 (W) cm force plate (BMS400600; AMTI, Watertown, USA) connected to a GEN-5 amplifier (AMTI, Watertown, USA) while simultaneously recording EEG. The force plate was synchronized with the EEG by recording ground reaction forces (Fx, Fy, Fz) and moments of force (Mx, My, Mz) using six auxiliary sensor inputs connected to the Sensor and Trigger Extension (Brain Products GmbH, Gilching, Germany). Each participant was instructed to perform three bipedal standing tasks in a self-selected, natural posture while wearing shoes (47) for three times 20 s each: quiet stance with eyes open (Stance-EO), quiet stance with eyes closed (Stance-EC), and narrow stance with feet together and eyes open (Narrow-EO). With this fixed order progression, we ensured standardization and accounted for the transition from simple to complex tasks, which is particularly important for MS populations to prevent overwhelming participants early in the protocol (47). To minimize potential effects of fatigue, participants were offered rest breaks at any point during the protocol.

### 2.4 Data preprocessing and analysis

COP analyses were carried out in Python (version 3.13.9) using custom written code along with functions from the MNE-Python toolbox (48). For each condition, the three trials were concatenated prior to analysis. First, data were digitally low-pass filtered by a fourth order zero-lag Butterworth filter with a cut-off frequency of 10 Hz, and signals were resampled at 50 sample points/s, consistent to other studies using AMTI force plates (6, 49). The following COP parameters were calculated for each condition: path length (total length of the COP trajectory, in cm), amplitude in the antero-posterior (AP) and medio-lateral (ML) direction (range, defined as maximum minus minimum displacement, in cm), average speed (path length divided by trial duration, in cm/s), and root mean square (RMS) displacement in the AP and ML directions as well as a combined total RMS (each computed as the standard deviation of the respective COP signal around its mean; total RMS = √(RMS_AP^2^ + RMS_ML^2^); in cm), serving as an indicator of overall postural sway magnitude. These parameters are consistent with established measures in posturographic research (47).

EEG data preprocessing was performed using BrainVision Analyzer 2.3 (Brain Products GmbH, Gilching, Germany). Continuous EEG data were band-pass filtered (2 to 70 Hz) using a second order zero-phase IIR Butterworth filter with a notch filter (50 Hz). Based on visual inspection, bad channels were interpolated (0.14 ± 0.36 channels; max = 2 of 32 channels) using splines (order: 4; maximum degree: 10; lambda: 1E-05). After re-referencing the EEG data to the average across all channels, we separated the resting-state data into eyes open and eyes closed and separated the balance tasks from each other. For each balance condition, the three 20-s trials were concatenated into a single continuous 60-s recording. Subsequently, continuous recordings were segmented into epochs of 1 s with an overlap of 0.5 s. A semi-automatic artefact rejection was used (gradient <50 μV/ms; maximal allowed difference of 100 μV in 10 ms intervals; maximal/minimal amplitude −200 to 200 μV; lowest allowed activity of 0.5 μV in 100 ms intervals). Trials exceeding these limits were excluded after visual inspection (resting-state: 29 ± 22.2, resulting in 211 ± 26.3 retained segments; balance tasks: 17 ± 10.7, resulting in 108 ± 16.3 retained segments). Subsequently, independent component analysis (ICA) was performed, and components were visually inspected with respect to their topography and their time-series. Systematic artifacts such as eye blinks, cardiac artefacts or muscle activity were removed (7.3 ± 1.2 components; max = 8 of 32 components). In a final step, the signal was visually inspected again for residual artifacts. The number of rejected epochs, removed ICA components and retained segments during preprocessing were similar between conditions and groups (see Supplementary Table S1). To analyze the power spectral density (PSD) and aperiodic neural activity, we exported the preprocessed data to Python (version 3.13.9) and used custom-written code based on MNE-Python (48). Electrodes were grouped into five regions of interest (ROI): frontal (F3, Fz, F4), fronto-central (C3, Cz, C4, FC1, FC2), centro-parietal (CP1, CP2, P3, Pz, P4), occipital (O1, Oz, O2), and global (all 32 electrodes). This was based on prior research implicating fronto-central regions in error detection during postural control (25, 31) and centro-parietal regions in sensorimotor integration (23, 24). Furthermore, an occipital and a global ROI were additionally included to capture the widespread cortical alterations reported in MS (32, 42). For each condition and ROI, PSD was estimated using the multitaper method (50) with a bandwidth of 2.0 Hz. Mean power was extracted within theta (4–8 Hz), alpha (8–13 Hz), beta (13–30 Hz), and gamma (30–45 Hz) frequency bands. Aperiodic neural activity was additionally characterized using the specparam algorithm (formerly FOOOF) (51), which decomposes the EEG power spectrum into periodic (oscillatory) and aperiodic components. The aperiodic component was parameterized across a frequency range of 3–40 Hz, consistent with previous electrophysiological analyses in MS (52), using a fixed aperiodic mode, peak width limits of 1–8 Hz (defining the minimum and maximum acceptable peak width in Hz), and a maximum of 6 peaks. The specparam model demonstrated good fitting performance across both groups and regions (*R*^2^ range: 0.977–0.990), confirming reliable spectral decomposition (for detailed values see Supplementary Table S2).

### 2.5 Statistical analysis

Statistical analyses were performed using Python (version 3.13.9) with the following packages: statsmodels (53), SciPy (54), pandas (55), and NumPy (56). COP variables were analyzed using Linear Mixed Models (LMM). Following Wilkinson notation (57), the LMM structure for all outcome variables was specified as: Outcome ∼ group * condition + (1∣participant) where group (MS vs. healthy controls), condition (Stance-EC, Narrow-EO; reference: Stance-EO), and their interactions are fixed effects, and (1∣participant) represents a random intercept per participant; random slopes were not modelled given the limited sample size. A sensitivity analysis was conducted by excluding the Stance-EC trial of one MS patient whose postural sway, while plausible given MS-related balance impairment, was exceptionally high. Models were fitted using restricted maximum likelihood (REML) for parameter estimation and semi-partial *R*^2^ computation. For likelihood ratio tests (LRT), models were refitted using maximum likelihood (ML). Main effects of condition and group × condition interactions were assessed via LRT (χ^2^, df = 2) comparing nested models. Effect sizes are reported as semi-partial R^2^, calculated as the proportional reduction in residual variance upon inclusion of each predictor via model comparison, with regression coefficients (*β*) and 95% confidence intervals. Effect size interpretation followed conventional benchmarks for regression-based effect sizes (small: 0.02, medium: 0.13, large: 0.26) (58). Prior to statistical analysis of EEG variables, frequency band power values (theta, alpha, beta, gamma) were log-transformed to approximate normality, consistent with previous EEG balance research (25, 30). Aperiodic parameters (exponent, offset) were not log-transformed, as these are dimensionless parameters estimated in log–log space by the specparam algorithm (51). Resting-state EEG group differences were assessed using two-sided Mann–Whitney U tests, computed separately for the eyes-open and eyes-closed conditions. Effect sizes are reported as rank-biserial correlation *r* (59). Stance EEG variables were baseline-corrected by subtracting the corresponding log-transformed resting-state condition prior to analysis (e.g., Stance-EO – resting-EO), thereby isolating task-induced cortical activity from individual baseline differences. Positive values reflect task-induced power increases relative to the resting baseline, whereas negative values reflect task-induced power decreases. This approach also controls for the well-known effect of eye state on EEG activity (e.g., alpha enhancement with eyes closed) (60, 61), ensuring that condition differences reflect postural demands rather than visual input per se. Baseline-corrected EEG values entered LMMs with the same fixed- and random-effects structure as the COP analysis. To control for multiple comparisons across variables, *p*-values were corrected using the Benjamini-Hochberg false discovery rate (FDR) procedure (62). Separate FDR correction families were defined for each effect type: COP analysis (3 families: group, condition, group × condition; *n* = 7 variables each); resting EEG (2 families: eyes-open, eyes-closed; *n* = 30 variables each); stance EEG (3 families: group, condition, group × condition; *n* = 30 variables each). Omnibus effects of condition and group × condition were assessed via likelihood-ratio tests (*df* = 2), with FDR-corrected *q*-values reported alongside uncorrected *p*-values for all analyses. Individual contrasts decomposing these omnibus effects are reported uncorrected.

## 3 Results

### 3.1 Posturography

MS patients demonstrated greater postural sway compared to healthy controls, with uncorrected group differences emerging for amplitude ML, RMS total, and RMS ML (semi-partial *R*^2^ range: 0.032–0.038; *p* = 0.026–0.032; all *q* = 0.076), reflecting a predominance of medio-lateral postural instability (see Table 2; Figure 1). FDR-corrected main effects of condition emerged for five variables (amplitude AP, amplitude ML, RMS total, RMS AP, and RMS ML; semi-partial *R*^2^ range: 0.358–0.585; all *p* < 0.001; *q* < 0.001–0.001; see Table 2). However, post-hoc contrasts revealed weak individual effects, with only amplitude AP and RMS AP showing a significant difference during Narrow-EO compared to Stance-EO (*p* = 0.005 and *p* = 0.012, respectively; see Table 3). Additionally, FDR-corrected group × condition interactions emerged for five variables in the overall interaction (amplitude AP, amplitude ML, RMS total, RMS AP, and RMS ML; semi-partial *R*^2^ range: 0.225–0.441; *p* < 0.001–0.009; *q* < 0.001–0.013; see Table 2). Individual contrasts revealed nominally significant interactions in all seven variables in the group × Stance-EC contrast (path length, amplitude AP, amplitude ML, average speed, RMS total, RMS AP, and RMS ML; semi-partial *R*^2^ range: 0.124–0.282; *p* = 0.001–0.033; see Table 3) and for three variables in the group × Narrow-EO contrast (amplitude AP, RMS AP, and RMS total; semi-partial R^2^ range: 0.205–0.448; *p* < 0.001–0.006; see Table 3). All interaction effects indicate that MS patients exhibited substantially greater postural destabilization than healthy controls when visual input was removed and when the base of support was reduced (see Table 3; Figure 1). An additional sensitivity analysis excluding the Stance-EC trial of one MS patient who exhibited exceptionally high postural sway (path length approximately 3 times the MS group mean) confirmed the robustness of these findings across both contrasts, with effect sizes remaining comparable or larger (see Supplementary Table S3).

**Table 2:** Linear mixed model results for center of pressure (COP) variables across stance conditions.

Variable	Group			Condition			Group × Condition		
	*p*	*q*	*sp-R^2^*	*p*	*q*	*sp-R^2^*	*p*	*q*	*sp-R^2^*
Path length	0.676	0.676	0.000	0.051	0.051	0.130	0.088	0.088	0.089
Amplitude AP	0.057	0.082	0.025	<0.001***	<0.001***	0.585	<0.001***	<0.001***	0.441
Amplitude ML	0.032*	0.076	0.038	<0.001***	0.001**	0.358	0.009**	0.013*	0.225
Average speed	0.676	0.676	0.000	0.051	0.051	0.130	0.088	0.088	0.089
RMS total	0.027*	0.076	0.032	<0.001***	<0.001***	0.403	0.003**	0.007**	0.285
RMS AP	0.058	0.082	0.029	<0.001***	<0.001***	0.540	0.002**	0.007**	0.304
RMS ML	0.026*	0.076	0.035	<0.001***	<0.001***	0.379	0.009**	0.013*	0.228

*p*, uncorrected *p*-value; *q*, Benjamini-Hochberg FDR-corrected *p*-value, applied separately for each effect type (Group, Condition, Group × Condition) across all 7 variables; sp-R^2^, semi-partial *R*^2^; significant values are shown in bold with *p/*q* < 0.05, **p/*q* < 0.01, and ***p/*q* < 0.001. Individual contrasts are reported in Table 3.

**Figure 1:**
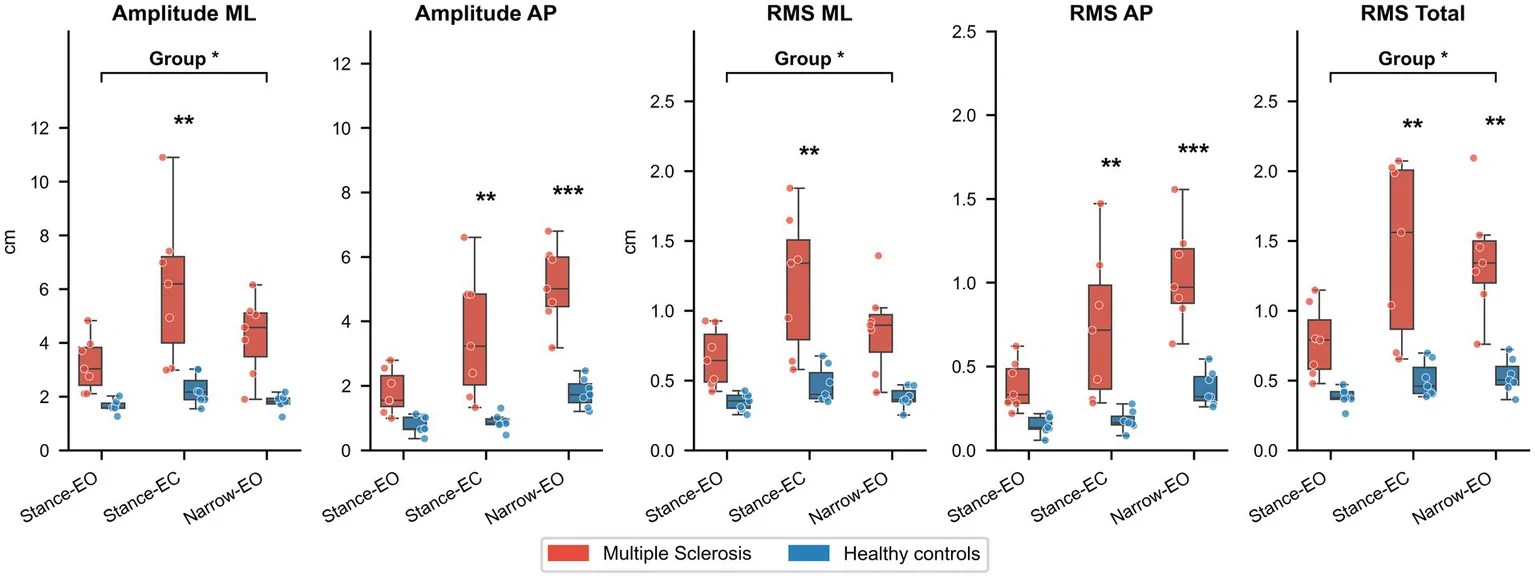
Center of pressure (COP) parameters in individuals with MS (red) and healthy controls (blue) across three balance conditions. Boxplots with individual data points (shown as circles) were chosen to transparently represent raw data distributions given the small sample size. Boxes show median and interquartile range. Variables with uncorrected main effects of group and exemplary group × condition interactions are displayed (for complete results see Supplementary Figure S1). “Group” brackets indicate an uncorrected main effect of group; asterisks above individual conditions indicate uncorrected group × condition contrasts (*p < 0.05, **p < 0.01, ***p < 0.001). AP, antero-posterior; ML, medio-lateral; RMS, root mean square.

**Table 3:** Individual contrast estimates from linear mixed model for center of pressure (COP) variables.

Variable	Condition: Stance-EC vs. Stance-EO			Condition: Narrow-EO vs. Stance-EO			Group × Stance-EC			Group × Narrow-EO		
	*β*	*p*	*sp-R^2^*	*β*	*p*	*sp-R^2^*	*β*	*p*	*sp-R^2^*	*β*	*p*	*sp-R^2^*
Path length	+3.040	0.768	0.154	+4.196	0.684	0.061	+31.002	0.033*	0.124	+16.909	0.245	0.014
Amplitude AP	+0.093	0.794	0.194	+0.999	0.005**	0.598	+1.652	0.001**	0.282	+2.315	<0.001***	0.448
Amplitude ML	+0.599	0.250	0.373	+0.149	0.774	0.038	+2.258	0.002**	0.252	+0.900	0.222	0.019
Average speed	+0.152	0.768	0.154	+0.210	0.684	0.061	+1.550	0.033*	0.124	+0.845	0.245	0.014
RMS total	+0.122	0.287	0.363	+0.149	0.195	0.339	+0.534	0.001**	0.282	+0.443	0.006**	0.205
RMS AP	+0.027	0.763	0.167	+0.221	0.012*	0.555	+0.325	0.009**	0.188	+0.439	<0.001***	0.313
RMS ML	+0.121	0.202	0.394	+0.030	0.752	0.047	+0.416	0.002**	0.256	+0.173	0.198	0.026

Individual contrast *p*-values are reported uncorrected, as they decompose the FDR-corrected omnibus tests reported in Table 2. *β*, unstandardized regression coefficient; sp-*R*^2^, semi-partial R^2^; significant values (*p* < 0.05) are shown in bold with *p < 0.05, **p < 0.01, and ***p < 0.001.

### 3.2 EEG

No electrocortical effects survived FDR correction. Prior to correction, resting-state EEG showed no group differences, except for elevated occipital theta power in MS patients during resting-EO (*U* = 43.0, *p* = 0.017, *q* = 0.524, *r* = −0.76; for complete results see Supplementary Table S4). During stance, no main effects of group were found. However, several uncorrected main effects of condition were observed for theta, alpha, and beta power across fronto-central, centro-parietal, and global regions (semi-partial *R*^2^ range: 0.205–0.288; *p* = 0.009–0.040; *q* = 0.190–0.201; see Table 4), with regression coefficients indicating that baseline-corrected power was more negative relative to Stance-EO. Notably, for these six variables, neither individual contrast reached significance when tested separately (Stance-EC vs. Stance-EO and Narrow-EO vs. Stance-EO, all *p* > 0.05; see Table 5). Uncorrected group × condition interactions emerged for eight frequency band variables (frontal theta and alpha; fronto-central theta; centro-parietal theta and beta; global theta, alpha, and beta; semi-partial *R*^2^ range: 0.193–0.318; *p* = 0.005–0.049; *q* = 0.134–0.163; see Table 4) and one aperiodic variable (centro-parietal offset, semi-partial *R*^2^ = 0.199, *p* = 0.045; *q* = 0.163; see Table 4). For frequency band variables, these interactions were driven exclusively by the group × Stance-EC contrast, with no group × Narrow-EO effects (see Figure 2), indicating that individuals with MS showed a consistently greater task-induced power decrease during EC stance relative to their resting baseline compared to healthy controls (see Table 5). For the centro-parietal aperiodic offset, the overall interaction was nominally significant (*p* = 0.045), but neither individual contrast reached significance (see Table 5).

**Table 4:** Significant results from linear mixed models for stance EEG.

ROI	Parameter	Group			Condition			Group × Condition		
		*p*	*q*	*sp-R^2^*	*p*	*q*	*sp-R^2^*	*p*	*q*	*sp-R^2^*
Frequency band power										
Frontal	Theta	0.352	0.992	0.010	0.113	0.283	0.144	0.013*	0.134	0.265
	Alpha	0.491	0.992	0.005	0.084	0.279	0.162	0.048*	0.163	0.195
Fronto-Central	Theta	0.482	0.992	0.007	0.040*	0.201	0.205	0.039*	0.163	0.207
	Alpha	0.904	0.992	0.000	0.014*	0.190	0.261	0.068	0.169	0.175
Centro-Parietal	Theta	0.205	0.992	0.018	0.160	0.370	0.123	0.046*	0.163	0.198
	Alpha	0.417	0.992	0.007	0.032*	0.194	0.217	0.054	0.163	0.188
	Beta	0.201	0.992	0.007	0.061	0.230	0.181	0.013*	0.134	0.267
Global	Theta	0.959	0.992	0.000	0.025*	0.190	0.231	0.020*	0.147	0.245
	Alpha	0.804	0.992	0.001	0.009**	0.190	0.288	0.049*	0.163	0.193
	Beta	0.947	0.992	0.000	0.025*	0.190	0.232	0.005**	0.134	0.318
Aperiodic activity										
Centro-Parietal	Offset	0.144	0.992	0.032	0.853	0.898	0.011	0.045*	0.163	0.199

ROI, region of interest; *p*, uncorrected *p*-value; *q*, Benjamini-Hochberg FDR-corrected *p*-value, applied separately for each effect type (Group, Condition, Group × Condition) across all 30 variables; sp-*R*^2^, semi-partial *R*^2^; significant values are shown in bold with *p/*q* < 0.05, **p/*q* < 0.01, and ***p/*q* < 0.001. Individual contrasts are reported in Table 5.

**Table 5:** Individual contrast estimates from linear mixed models for stance EEG.

ROI	Parameter	Condition						Group × Condition					
		*β* (EC vs. EO)	*p*	*sp-R^2^*	*β* (Narrow vs. EO)	*p*	*sp-R^2^*	*β* (Grp×EC)	*p*	*sp-R^2^*	*β* (Grp×Narrow)	*p*	*sp-R^2^*
Frequency band power													
Frontal	Theta	0.050	0.704	0.138	0.000	0.999	0.015	−0.521	0.005**	0.250	−0.130	0.482	0.020
	Alpha	0.000	0.998	0.160	−0.070	0.665	0.068	−0.547	0.017*	0.191	−0.198	0.388	0.030
Fronto-Central	Theta	−0.044	0.678	0.203	−0.080	0.453	0.038	−0.331	0.029*	0.166	−0.005	0.972	0.000
	Alpha	−0.094	0.437	0.258	−0.145	0.230	0.118	−0.354	0.038*	0.152	−0.047	0.781	0.003
Centro-Parietal	Theta	−0.020	0.859	0.121	−0.107	0.343	0.021	−0.283	0.076	0.116	0.087	0.584	0.012
	Alpha	−0.068	0.632	0.216	−0.137	0.334	0.086	−0.435	0.030*	0.164	−0.060	0.766	0.004
	Beta	0.004	0.948	0.128	−0.130	0.038*	0.154	−0.203	0.022*	0.178	0.043	0.626	0.010
Global	Theta	−0.037	0.727	0.225	−0.057	0.594	0.032	−0.386	0.011*	0.212	−0.042	0.781	0.003
	Alpha	−0.105	0.411	0.288	−0.107	0.402	0.095	−0.418	0.021*	0.182	−0.106	0.558	0.014
	Beta	0.001	0.988	0.220	−0.078	0.248	0.131	−0.304	0.002**	0.296	−0.065	0.501	0.019
Aperiodic activity													
Centro-Parietal	Offset	0.047	0.433	0.009	−0.054	0.371	0.008	−0.138	0.105	0.099	0.065	0.443	0.024

Only variables with at least one uncorrected significant effect (*p* < 0.05) are shown. ROI, region of interest; *β*, unstandardized regression coefficient (log-ratio units for frequency band power; raw units for aperiodic parameters); *β* (EC vs. EO) and *β* (Narrow vs. EO), individual condition contrasts against Stance-EO; sp-*R*^2^, semi-partial *R*^2^; significant values (*p* < 0.05) are shown in bold with *p < 0.05, **p < 0.01, and ***p < 0.001.

**Figure 2:**
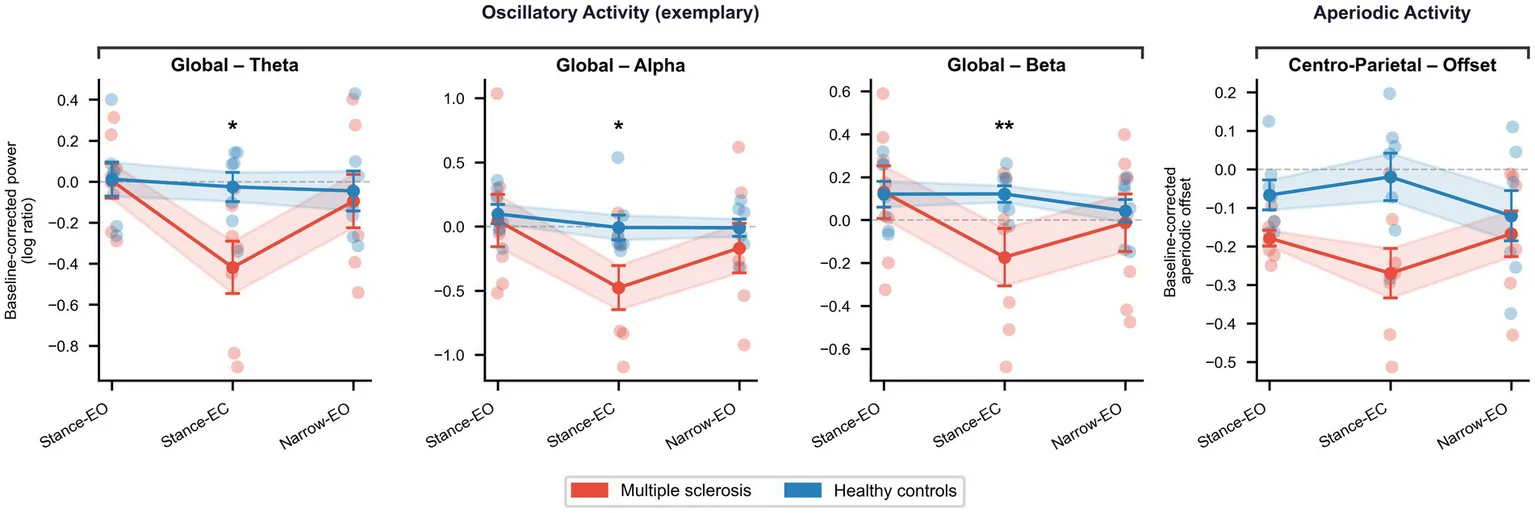
Group × Stance-EC interactions for oscillatory and aperiodic EEG activity in individuals with MS (red) and healthy controls (blue). Individual participant values (semi-transparent dots) are shown alongside mean ± SEM line plots, with baseline-corrected values emphasizing task-induced changes relative to resting state. Oscillatory activity is exemplarily displayed for the global region of interest. Results for all interactions across all regions of interest are provided in Supplementary Figure S2. Baseline correction was performed by subtracting the corresponding resting-state condition (e.g., Stance-EO − resting-EO). Asterisks indicate uncorrected group × Stance-EC contrasts (*p < 0.05, **p < 0.01, ***p < 0.001). The centro-parietal aperiodic offset panel displays no significance markers as the overall group × condition interaction (*p* = 0.045) was not reflected by significant individual contrasts.

## 4 Discussion

This exploratory study investigated oscillatory and aperiodic brain activity in individuals with MS compared to age- and sex-matched healthy controls during static balance tasks on a force plate. Postural demands were manipulated through visual deprivation and a reduction in base of support. MS patients demonstrated greater postural sway than healthy controls, with group differences predominantly in medio-lateral measures, and a robust destabilization across all COP variables during standing with eyes-closed. Enhanced visual reliance for postural control seems to be reflected in uncorrected EEG findings suggesting a task-related cortical desynchronization during eyes-closed stance only. This is consistent with the hypothesis that MS involves heightened reliance on visual input for balance control.

### 4.1 Postural instability in MS

Individuals with MS demonstrated greater postural sway compared to healthy controls across all balance conditions. These findings are consistent with previous reports demonstrating balance impairments in MS patients even under less demanding standing conditions (7). The strongest group effects emerged for medio-lateral control, which is a clinically significant finding given that lateral sway is a primary predictor of fall risk (17). Although preliminary group differences in medio-lateral measures did not survive FDR correction, postural instability became substantially more pronounced under challenging postural conditions. FDR-corrected group × condition interactions with moderate to large effect sizes emerged across all seven COP variables for Stance-EC (semi-partial *R*^2^ = 0.124–0.282) and three variables for Narrow-EO (semi-partial *R*^2^ = 0.205–0.448). Interestingly, the Narrow-EO interactions were confined to antero-posterior measures, reflecting distinct neuromuscular control strategies required during narrow stance compared to regular side-by-side stance (63). However, the more pronounced destabilization during Stance-EC suggests that visual deprivation is a particularly potent destabilizing factor in MS, consistent with prior studies highlighting the heavy reliance of MS patients on visual feedback for postural control (11, 19). This phenomenon may be driven by functional impairment within the somatosensory system, leading to deficits in the regulation of postural control (11). This suggests that the observed balance instability is not merely a behavioral preference for vision, but rather a functional failure of alternative sensory modalities to adequately compensate for the loss of visual input. However, this interpretation requires direct assessment of somatosensory or vestibular function to be confirmed.

### 4.2 The role of visual information in MS-related cortical responses

During resting state, the only uncorrected cortical group difference was elevated occipital theta power in MS patients during the eyes-open condition. This may reflect a visual cortex dysfunction, consistent with findings linking increased occipital beta activity to MS-related structural impairments of the visual system, such as reduced retinal nerve fiber layer thickness (64). This raises the possibility that occipital cortical excitability, here reflected by heightened theta power, is chronically altered in MS. Given that this was an isolated finding, this interpretation remains highly speculative and warrants replication in larger samples. In general, the present findings do not provide consistent evidence for widespread resting-state abnormalities, which contrasts with other studies that reported heterogeneous resting-state EEG abnormalities in MS, mainly characterized by an increased and more diffuse slow-wave activity (32). However, the overall similarity in resting-state activity between groups supports the use of the baseline correction for the stance analyses. This makes it more likely that the cortical changes during balance tasks reflect task-specific modulations driven by postural demands rather than pre-existing group differences at rest. Notably, most existing EEG balance studies report absolute power rather than baseline-corrected values, which should be considered when comparing the present findings with the existing literature.

During stance, uncorrected group differences emerged exclusively through group × Stance-EC interactions but not during Narrow-EO, suggesting that cortical responses to postural challenges were modulated by MS primarily when visual information was withdrawn. Relative to their matched eyes-open resting baseline, MS patients exhibited a greater task-induced suppression of oscillatory power upon eye closure. This effect, although it did not survive FDR correction, spanned theta, alpha, and beta bands across frontal, fronto-central, centro-parietal, and global regions, while gamma power remained unaffected, aligning with the distributed cortical networks thought to underlie postural control (21, 22, 24). The fact that these differences emerged in the eyes-closed condition is in line with the clinical observation that individuals with MS rely more heavily on visual feedback for balance (11, 19). This reliance was found even at comparatively low disability levels, with a mean EDSS of 1.9 ± 1.0 (65), and is particularly evident in the presence of increased somatosensory deficits (19). Although not directly assessed, such deficits are likely prevalent in the present sample given the moderate-to-severe disability level (median EDSS: 5.5, IQR: 4.5–6.0). The absence of cortical group differences during Narrow-EO, despite large COP group differences, further suggests that postural challenge alone is insufficient; rather, visual deprivation may be a critical factor for cortical modulations. However, it is important to emphasize that interpreting this pattern as increased visual reliance is based on the observed behavioral and cortical findings rather than on a direct assessment of the sensory contributions to balance. The exploratory cortical desynchronization might therefore be a potential neural correlate of postural control under visually challenging conditions rather than a marker of sensory reweighting.

### 4.3 Enhanced task-related power reductions of oscillatory activity

The task-related cortical desynchronization is first reflected by uncorrected evidence of alpha suppression upon eye closure in MS patients. This is consistent with previous studies in healthy individuals reporting decreased alpha power with increasing balance task difficulty (23–25). This effect may reflect heightened cortical engagement in sensory processing, as alpha oscillations are proposed to index the active gating of task-irrelevant input (26, 27). This suggests that MS patients recruited more cortical resources when visual input was removed. Second, the apparent beta power reduction mirrors findings by Sipp et al. (28), who reported lower beta power during walking on a balance beam compared to treadmill walking. Since beta desynchronization is generally associated with increased sensorimotor processing (29), the greater beta suppression in MS patients during Stance-EC further highlights increased sensorimotor cortical engagement specifically when visual input is unavailable. However, given that beta power during continuous static balance tasks has received limited investigation to date (23), this finding provides a preliminary basis for understanding beta dynamics in balance, although further studies are needed to substantiate this interpretation. Interestingly, the present EEG analyses suggest a task-specific theta reduction in MS patients, which is in contrast to previous studies reporting theta increases with higher postural demands (24, 25, 30). This increased theta activity is linked to error detection and processing in fronto-central regions (28, 31). Importantly, Hülsdünker et al. (25, 30) employed an actively oscillating platform imposing continuous mechanical destabilization, and Gebel et al. (24) increased mechanical instability through progressive reduction of a balance board’s pivot surface. This means both approaches required continuous reactive motor corrections to counteract external mechanical perturbations, indicating theta oscillations may be specifically recruited when balance errors are continuously generated by external mechanical forces. This is consistent with Edwards et al. (15, 16), who observed no consistent theta modulation during static balance and proposed that opposing event-sensitive theta changes may cancel out under steady-state conditions, unlike mechanically destabilizing paradigms. This may more broadly explain the minimal cortical modulation observed across all frequency bands in healthy controls, potentially reflecting insufficient task difficulty to produce robust cortical responses under stable surface conditions.

### 4.4 Preliminary evidence for altered aperiodic broadband excitability

Beyond oscillatory activity, the present study is, to our knowledge, the first to integrate aperiodic parameters into the analysis of postural control in MS, providing a more holistic perspective on neural dynamics. However, only an uncorrected and nominally significant group × condition interaction was observed for the centro-parietal aperiodic offset. This may reflect diminished broadband cortical excitability in sensorimotor regions (37, 38). Importantly, as individual condition contrasts did not reach significance, potentially due to the limited sample size, this interpretation remains speculative. Notably, no significant effects were observed for the aperiodic exponent, despite it being the more frequently investigated aperiodic parameter in MS (39–41). This may suggest that E:I balance, as indexed by the exponent, is less sensitive to postural demands than broadband cortical excitability as indexed by the offset. However, this distinction warrants replication in larger samples. The present findings nonetheless provide preliminary indication that the aperiodic offset may be sensitive to postural demands and their interaction with MS-related cortical alterations.

### 4.5 Limitations and clinical implications

The results of this pilot study and the derived interpretations should be considered in light of several limitations. First, the modest sample size (*n* = 14) limits statistical power and generalizability. Due to excessive movement artifacts in the EEG during balance tasks, we had to exclude 9 of 23 participants (39%). Although no EEG effects survived FDR correction and all cortical findings should therefore be interpreted as preliminary, the observed effects were of moderate to large magnitude for both posturographic interaction effects (semi-partial *R*^2^ = 0.225–0.441) and EEG interaction effects (semi-partial *R*^2^ = 0.193–0.318). Effect sizes of this magnitude should nonetheless be interpreted cautiously, as effects estimated in small samples are prone to overestimation, and replication in larger cohorts is essential. Second, our preprocessing of the EEG data reduced the likelihood of systematic artifact differences between groups and conditions (see Supplementary Table S1). However, residual movement-related contamination remains a general limitation of mobile EEG and cannot be definitively excluded here, particularly during Stance-EC, the condition with the greatest postural instability. Future studies should therefore incorporate sway-related covariates to determine whether these effects persist after accounting for movement magnitude. Third, manipulating the availability of vision isolates the behavioral consequences of visual deprivation, but not the sensory mechanisms behind them. Impaired somatosensory integration, vestibular dysfunction, cerebellar involvement, or optic pathway impairment could each contribute to the observed effects (66). Disentangling these would require paradigms that directly quantify sensory contributions to balance. Fourth, the cross-sectional design precludes causal inferences about the relationship between oscillatory or aperiodic brain activity and balance control. Therefore, future longitudinal studies should investigate whether improvements in postural control following interventions are accompanied by modulation of the observed EEG parameters. Physical exercise represents a particularly promising intervention in this context (67). Studies in healthy individuals have demonstrated effects of physical training on oscillatory activity including increases in alpha and beta power (68) and modulations in aperiodic activity alongside improvements in cognitive function (69). Whether comparable training-induced modulation of electrocortical activity occurs in MS patients and relates to improvements in postural control is currently under investigation in a large-scale multi-center study (44). If confirmed, oscillatory and aperiodic EEG activity could represent promising neurophysiological markers of postural control for impairments and rehabilitation in MS.

### 4.6 Conclusion

Individuals with MS demonstrated greater postural sway compared to healthy controls, with uncorrected evidence of a medio-lateral predominance, most pronounced during standing with eyes closed. While uncorrected EEG findings indicated task-related cortical reductions in theta, alpha, and beta power specifically upon eye closure in MS patients, healthy controls showed minimal cortical modulation across conditions. These preliminary findings in a small sample are consistent with an increased reliance on visual information for postural control, accompanied by a task-related cortical desynchronization. We propose that future confirmatory studies in larger samples investigate whether the oscillatory and aperiodic patterns observed here are reliably associated with balance deficits under visually challenging conditions.

## Data Availability

The raw data supporting the conclusions of this article will be made available by the authors, without undue reservation.
