# Supplementary material for: Increased visual reliance for postural control in multiple sclerosis: preliminary evidence from task-related cortical desynchronization
Source: Front Neurol. 2026 Sep 30;17:1882165. doi: 10.3389/fneur.2026.1882165 (PMC13661309; doi:10.3389/fneur.2026.1882165)
Supplement: Supplementary file 1 [file Supplementary_file_1.DOCX]

Supplementary Material

**Supplementary Table S1: EEG data quality separated by group and condition**

| **Preprocessing step** | **Stance-EO** | | **Stance-EC** | | **Narrow-EO** | |
| --- | --- | --- | --- | --- | --- | --- |
|  | **MS** | **HC** | **MS** | **HC** | **MS** | **HC** |
| # Removed segments | 13.29 (±3.15) | 17.43 (±8.68) | 14.29 (±8.58) | 12.86 (±6.46) | 26.00 (±9.22) | 17.00 (±12.77) |
| # Kept segments | 101.86 (±12.68) | 115.57 (±17.80) | 105.29 (±13.15) | 115.14 (±12.39) | 98.00 (±4.14) | 114.29 (±17.70) |
| # ICA removed | 7.89 (±0.38) | 7.71 (±0.76) | 8.00 (±1.15) | 7.71 (±1.80) | 8.00 (±0.00) | 8.00 (±0.76) |

*Values represent mean (± SD). MS = Multiple sclerosis; HC = Healthy controls; EO = eyes open; EC = eyes closed. # Removed segments = artifact-rejected epochs; # Kept segments = retained epochs; # ICA removed = independent components removed.*

**Supplementary Table S2. specparam model fitting performance across regions of interest and groups.**

| **Region of interest** | **Multiple Sclerosis (R²)** | **Healthy controls (R²)** |
| --- | --- | --- |
| Frontal | 0.979 ± 0.016 | 0.977 ± 0.041 |
| Fronto-Central | 0.986 ± 0.009 | 0.986 ± 0.006 |
| Centro-Parietal | 0.990 ± 0.004 | 0.986 ± 0.008 |
| Occipital | 0.985 ± 0.007 | 0.982 ± 0.013 |
| Global | 0.987 ± 0.008 | 0.985 ± 0.011 |

*R² = coefficient of determination reflecting specparam model fit quality. Values represent mean ± standard deviation across participants.*

**Supplementary Figure S1: All COP parameters across balance conditions.**

*All center of pressure (COP) parameters in individuals with multiple scleroris (MS; red) and healthy controls (blue) across three balance conditions. Boxplots with individual data points (shown as circles) were chosen to transparently represent raw data distributions. Boxes show median and interquartile range. Note that path length and average speed appear visually skewed due to one MS participant exhibiting exceptionally high postural sway during Stance-EC; a sensitivity analysis excluding this trial confirmed the robustness of all findings (see Supplementary Table S2). 'Group' brackets indicate a significant main effect of group; asterisks above individual conditions indicate significant group × condition contrasts (* p < .05, ** p < .01, *** p < .001). AP, antero-posterior; ML = medio-lateral; MS = multiple sclerosis; RMS = root mean square.*

**Supplementary Figure S2: All significant group × condition interactions for oscillatory and aperiodic EEG activity.**

*Mean baseline-corrected values (± SEM) with individual participant values (semi-transparent dots) are shown for individuals with MS (red) and healthy controls (blue). Upper two rows: frequency band power (log ratio); lower panel: aperiodic offset. Asterisks indicate significant Group × Stance-EC contrasts (* p < .05, ** p < .01, *** p < .001). FC, fronto-central; CP, centro-parietal; SEM = standard error of the mean.*

**Supplementary Table S3. Sensitivity analysis: Linear Mixed Model results excluding outlier during Stance-EC trial**

| **Variable** | **Group** | | | **Condition** | | | **Group × Condition** | | | **Group × Stance-EC** | | | **Group × Narrow-EO** | | | |
| --- | --- | --- | --- | --- | --- | --- | --- | --- | --- | --- | --- | --- | --- | --- | --- | --- |
|  | ***p*** | ***q*** | ***sp-R²*** | ***p*** | ***q*** | ***sp-R²*** | ***p*** | ***q*** | ***sp-R²*** | ***β*** | ***p*** | ***sp-R²*** | ***β*** | ***p*** | ***sp-R²*** |  |
| Path length | .418 | .418 | .000 | **< .001***** | **< .001***** | .435 | **.001**** | **.002**** | .371 | +13.641 | **.003**** | .261 | +16.909 | **< .001***** | .365 |  |
| Amplitude AP | .052 | .083 | .026 | **< .001***** | **< .001***** | .605 | **< .001***** | **.001**** | .458 | +1.448 | **.004**** | .236 | +2.315 | **< .001***** | .474 |  |
| Amplitude ML | **.006**** | **.042*** | .028 | **< .001***** | **< .001***** | .462 | **.004**** | **.004**** | .284 | +1.508 | **.001**** | .307 | +0.900 | **.037*** | .123 |  |
| Average speed | .418 | .418 | .000 | **< .001***** | **< .001***** | .435 | **.001**** | **.002**** | .371 | +0.682 | **.003**** | .261 | +0.845 | **< .001***** | .365 |  |
| RMS total | **.019*** | **.044*** | .028 | **< .001***** | **< .001***** | .447 | **.002**** | **.003**** | .307 | +0.423 | **.003**** | .248 | +0.443 | **.001**** | .283 |  |
| RMS AP | .059 | .083 | .030 | **< .001***** | **< .001***** | .548 | **.002**** | **.003**** | .300 | +0.300 | **.019*** | .153 | +0.439 | **< .001***** | .318 |  |
| RMS ML | **.012*** | **.043*** | .029 | **< .001***** | **< .001***** | .409 | **.013*** | **.013*** | .212 | +0.304 | **.003**** | .241 | +0.173 | .085 | .076 |  |

*Sensitivity analysis excluding the Stance-EC trial of one participant (outlying COP values). p/q = uncorrected p-value and Benjamini-Hochberg FDR-corrected p-value, applied separately for each effect type (group, condition, group × condition) across all 7 variables; β = unstandardized regression coefficient; sp-R² = semi-partial R². Individual contrasts (group × Stance-EC, group × Narrow-EO) decompose the omnibus interaction and are reported uncorrected. Significant values are shown in bold with * p/q < .05, ** p/q < .01, and *** p/q < .001.*

**Supplementary Table S4. Complete resting-state** **EEG group comparisons (Mann-Whitney U Tests)**

|  | **ROI** | **Parameter** | **MS (M ± SD)** | **Healthy (M ± SD)** | ***U*** | ***p*** | ***q*** | ***r*** |
| --- | --- | --- | --- | --- | --- | --- | --- | --- |
| Resting Eyes Open | frontal | theta | 5.59 ± 0.47 | 5.56 ± 0.47 | 24 | *1.000* | 1.000 | +0.02 |
|  |  | alpha | 5.75 ± 0.65 | 5.49 ± 0.68 | 29 | *.620* | 1.000 | -0.18 |
|  |  | beta | 4.97 ± 0.52 | 4.89 ± 0.33 | 29 | *.620* | 1.000 | -0.18 |
|  |  | gamma | 3.92 ± 0.67 | 3.91 ± 0.49 | 25 | *1.000* | 1.000 | -0.02 |
|  |  | exponent | 0.74 ± 0.35 | 0.77 ± 0.37 | 24 | *1.000* | 1.000 | +0.02 |
|  |  | offset | -9.12 ± 0.35 | -9.09 ± 0.45 | 19 | *.535* | 1.000 | +0.22 |
|  | fronto-central | theta | 5.60 ± 0.57 | 5.56 ± 0.60 | 25 | *1.000* | 1.000 | -0.02 |
|  |  | alpha | 5.72 ± 0.58 | 5.46 ± 0.84 | 27 | *.805* | 1.000 | -0.10 |
|  |  | beta | 4.82 ± 0.64 | 4.72 ± 0.44 | 24 | *1.000* | 1.000 | +0.02 |
|  |  | gamma | 3.67 ± 0.45 | 3.34 ± 0.26 | 37 | *.128* | .618 | -0.51 |
|  |  | exponent | 0.88 ± 0.25 | 1.06 ± 0.20 | 11 | *.097* | .584 | +0.55 |
|  |  | offset | -9.03 ± 0.30 | -8.87 ± 0.28 | 13 | *.165* | .618 | +0.47 |
|  | centro-parietal | theta | 5.95 ± 0.78 | 5.65 ± 0.74 | 30 | *.535* | 1.000 | -0.22 |
|  |  | alpha | 6.28 ± 0.69 | 5.80 ± 0.95 | 34 | *.259* | .778 | -0.39 |
|  |  | beta | 5.04 ± 0.74 | 4.86 ± 0.56 | 29 | *.620* | 1.000 | -0.18 |
|  |  | gamma | 3.52 ± 0.51 | 3.36 ± 0.26 | 28 | *.710* | 1.000 | -0.14 |
|  |  | exponent | 1.07 ± 0.18 | 1.09 ± 0.21 | 23 | *.902* | 1.000 | +0.06 |
|  |  | offset | -8.78 ± 0.36 | -8.79 ± 0.37 | 26 | *.902* | 1.000 | -0.06 |
|  | occiptal | **theta** | **6.45 ± 0.48** | **5.72 ± 0.59** | **43** | ***.017**** | **.524** | **-0.76** |
|  |  | alpha | 6.57 ± 0.47 | 5.72 ± 0.71 | 40 | *.053* | .530 | -0.63 |
|  |  | beta | 5.53 ± 0.74 | 4.99 ± 0.59 | 36 | *.165* | .618 | -0.47 |
|  |  | gamma | 4.30 ± 0.54 | 3.96 ± 0.19 | 40 | *.053* | .530 | -0.63 |
|  |  | exponent | 0.99 ± 0.23 | 0.85 ± 0.28 | 33 | *.318* | .794 | -0.35 |
|  |  | offset | -8.57 ± 0.36 | -8.94 ± 0.42 | 38 | *.097* | .584 | -0.55 |
|  | global | theta | 5.94 ± 0.55 | 5.69 ± 0.55 | 33 | *.318* | .794 | -0.35 |
|  |  | alpha | 6.16 ± 0.48 | 5.71 ± 0.74 | 35 | *.209* | .695 | -0.43 |
|  |  | beta | 5.11 ± 0.60 | 4.94 ± 0.38 | 28 | *.710* | 1.000 | -0.14 |
|  |  | gamma | 3.97 ± 0.56 | 3.90 ± 0.33 | 31 | *.456* | 1.000 | -0.27 |
|  |  | exponent | 0.87 ± 0.20 | 0.85 ± 0.24 | 27 | *.805* | 1.000 | -0.10 |
|  |  | offset | -8.89 ± 0.25 | -8.95 ± 0.31 | 26 | *.902* | 1.000 | -0.06 |
| Resting Eyes Closed | frontal | theta | 6.19 ± 0.70 | 5.85 ± 0.90 | 31 | *.456* | .927 | -0.27 |
|  |  | alpha | 6.57 ± 0.66 | 6.13 ± 1.15 | 29 | *.620* | .927 | -0.18 |
|  |  | beta | 5.06 ± 0.53 | 4.92 ± 0.67 | 28 | *.710* | .927 | -0.14 |
|  |  | gamma | 3.76 ± 0.67 | 3.56 ± 0.52 | 27 | *.805* | .966 | -0.10 |
|  |  | exponent | 1.00 ± 0.14 | 1.04 ± 0.23 | 18 | *.456* | .927 | +0.27 |
|  |  | offset | -8.77 ± 0.22 | -8.81 ± 0.46 | 21 | *.710* | .927 | +0.14 |
|  | fronto-central | theta | 6.02 ± 0.76 | 5.80 ± 0.98 | 26 | *.902* | 1.000 | -0.06 |
|  |  | alpha | 6.41 ± 0.71 | 6.01 ± 0.99 | 27 | *.805* | .966 | -0.10 |
|  |  | beta | 4.89 ± 0.66 | 4.89 ± 0.54 | 23 | *.902* | 1.000 | +0.06 |
|  |  | gamma | 3.39 ± 0.54 | 3.32 ± 0.25 | 28 | *.710* | .927 | -0.14 |
|  |  | exponent | 1.07 ± 0.13 | 1.10 ± 0.30 | 19 | *.535* | .927 | +0.22 |
|  |  | offset | -8.84 ± 0.29 | -8.81 ± 0.50 | 24 | *1.000* | 1.000 | +0.02 |
|  | centro-parietal | theta | 6.40 ± 0.95 | 5.88 ± 1.11 | 32 | *.383* | .927 | -0.31 |
|  |  | alpha | 7.16 ± 0.72 | 6.58 ± 1.30 | 31 | *.456* | .927 | -0.27 |
|  |  | beta | 5.30 ± 0.66 | 5.18 ± 0.74 | 29 | *.620* | .927 | -0.18 |
|  |  | gamma | 3.48 ± 0.55 | 3.42 ± 0.33 | 25 | *1.000* | 1.000 | -0.02 |
|  |  | exponent | 1.19 ± 0.22 | 1.11 ± 0.32 | 28 | *.710* | .927 | -0.14 |
|  |  | offset | -8.59 ± 0.41 | -8.74 ± 0.56 | 29 | *.620* | .927 | -0.18 |
|  | occiptal | theta | 7.04 ± 0.85 | 6.27 ± 1.05 | 34 | *.259* | .927 | -0.39 |
|  |  | alpha | 7.86 ± 0.84 | 7.13 ± 1.40 | 32 | *.383* | .927 | -0.31 |
|  |  | beta | 5.85 ± 0.67 | 5.54 ± 0.91 | 29 | *.620* | .927 | -0.18 |
|  |  | gamma | 4.29 ± 0.55 | 4.23 ± 0.56 | 25 | *1.000* | 1.000 | -0.02 |
|  |  | exponent | 1.13 ± 0.22 | 0.86 ± 0.39 | 37 | *.128* | .927 | -0.51 |
|  |  | offset | -8.33 ± 0.41 | -8.78 ± 0.61 | 38 | *.097* | .927 | -0.55 |
|  | global | theta | 6.54 ± 0.82 | 5.97 ± 1.02 | 36 | *.165* | .927 | -0.47 |
|  |  | alpha | 7.13 ± 0.65 | 6.47 ± 1.14 | 33 | *.318* | .927 | -0.35 |
|  |  | beta | 5.35 ± 0.55 | 5.11 ± 0.62 | 31 | *.456* | .927 | -0.27 |
|  |  | gamma | 3.91 ± 0.54 | 3.77 ± 0.35 | 28 | *.710* | .927 | -0.14 |
|  |  | exponent | 1.08 ± 0.12 | 0.97 ± 0.28 | 30 | *.535* | .927 | -0.22 |
|  |  | offset | -8.58 ± 0.34 | -8.81 ± 0.52 | 30 | *.535* | .927 | -0.22 |

*M ± SD = mean ± standard deviation (log-transformed power for frequency bands; raw units for aperiodic parameters). r = rank-biserial correlation (negative values indicate higher values in MS). q = Benjamini-Hochberg FDR-corrected p-value. Significant values (p < .05) are shown in bold. * p < .05. No effects survived FDR correction (all q > .05).*

**Supplementary Table S5. Complete LMM results for all stance EEG variables**

| ROI | Parameter | **Main effect: Group** | | | **Main effect: Condition** | | | **Interaction Group × Condition** | | |
| --- | --- | --- | --- | --- | --- | --- | --- | --- | --- | --- |
|  |  | ***p*** | ***q*** | ***sp-R²*** | ***p*** | ***q*** | ***sp-R²*** | ***p*** | ***q*** | ***sp-R²*** |
| frontal | theta | .352 | .992 | .010 | .113 | .283 | .144 | **.013*** | .134 | .265 |
|  | alpha | .491 | .992 | .005 | .084 | .279 | .162 | **.048*** | .163 | .195 |
|  | beta | .966 | .992 | .000 | .227 | .401 | .101 | .063 | .169 | .179 |
|  | gamma | .432 | .992 | .011 | .731 | .890 | .022 | .237 | .400 | .098 |
|  | exponent | .919 | .992 | .000 | .398 | .568 | .064 | .908 | .908 | .007 |
|  | offset | .716 | .992 | .001 | .218 | .401 | .103 | .425 | .554 | .059 |
| fronto-central | theta | .482 | .992 | .007 | **.040*** | .201 | .205 | **.039*** | .163 | .207 |
|  | alpha | .904 | .992 | .000 | **.014*** | .190 | .261 | .068 | .169 | .175 |
|  | beta | .691 | .992 | .003 | .112 | .283 | .145 | .270 | .426 | .089 |
|  | gamma | .713 | .992 | .002 | .612 | .798 | .035 | .878 | .908 | .009 |
|  | exponent | .825 | .992 | .001 | .787 | .893 | .017 | .449 | .561 | .056 |
|  | offset | .992 | .992 | .000 | .803 | .893 | .016 | .213 | .400 | .105 |
| centro-parietal | theta | .205 | .992 | .018 | .160 | .370 | .123 | **.046*** | .163 | .198 |
|  | alpha | .417 | .992 | .007 | **.032*** | .194 | .217 | .054 | .163 | .188 |
|  | beta | .201 | .992 | .007 | .061 | .230 | .181 | **.013*** | .134 | .267 |
|  | gamma | .855 | .992 | .000 | .106 | .283 | .148 | .671 | .746 | .028 |
|  | exponent | .287 | .992 | .014 | .908 | .908 | .007 | .208 | .400 | .106 |
|  | offset | .144 | .992 | .032 | .853 | .898 | .011 | **.045*** | .163 | .199 |
| occiptal | theta | .904 | .992 | .000 | .217 | .401 | .103 | .223 | .400 | .102 |
|  | alpha | .975 | .992 | .000 | .052 | .223 | .190 | .321 | .458 | .078 |
|  | beta | .710 | .992 | .002 | .296 | .467 | .083 | .645 | .744 | .031 |
|  | gamma | .543 | .992 | .008 | .868 | .898 | .010 | .747 | .801 | .021 |
|  | exponent | .232 | .992 | .020 | .361 | .542 | .070 | .311 | .458 | .080 |
|  | offset | .493 | .992 | .008 | .273 | .455 | .089 | .240 | .400 | .097 |
| global | theta | .959 | .992 | .000 | **.025*** | .190 | .231 | **.020*** | .147 | .245 |
|  | alpha | .804 | .992 | .001 | **.009**** | .190 | .288 | **.049*** | .163 | .193 |
|  | beta | .947 | .992 | .000 | **.025*** | .190 | .232 | **.005**** | .134 | .318 |
|  | gamma | .120 | .992 | .022 | .741 | .890 | .021 | .385 | .526 | .066 |
|  | exponent | .061 | .992 | .049 | .498 | .679 | .049 | .525 | .630 | .045 |
|  | offset | .196 | .992 | .033 | .221 | .401 | .102 | .192 | .400 | .111 |

*No effects survived FDR correction (all q > .05). ROI = region of interest; p/q = uncorrected p-value and Benjamini-Hochberg FDR-corrected p-value, applied separately for each effect type (Group, Condition, Group × Condition) across all 30 variables; sp-R² = semi-partial R². Significant values are shown in bold with * p/q < .05, ** p/q < .01, and *** p/q < .001.*

**Supplementary Table S6: Complete individual contrast estimates from LMM for stance EEG**

| **ROI** | **Parameter** | **Main effect: Condition** | | | | | | **Interaction Group × Condition** | | | | | |
| --- | --- | --- | --- | --- | --- | --- | --- | --- | --- | --- | --- | --- | --- |
|  |  | ***β* (EC vs. EO)** | ***p*** | ***sp-R²*** | ***β* (Narrow vs. EO)** | ***p*** | ***sp-R²*** | ***β*  (Grp × EC)** | ***p*** | ***sp-R²*** | ***β*  (Grp × Narrow)** | ***p*** | ***sp-R²*** |
| frontal | theta | 0.050 | .704 | .138 | 0.000 | .999 | .015 | -0.521 | **.005**** | .250 | -0.130 | .482 | .020 |
|  | alpha | 0.000 | .998 | .160 | -0.070 | .665 | .068 | -0.547 | **.017*** | .191 | -0.198 | .388 | .030 |
|  | beta | 0.076 | .533 | .062 | -0.068 | .577 | .090 | -0.394 | **.023*** | .177 | -0.157 | .364 | .033 |
|  | gamma | 0.268 | .189 | .002 | 0.054 | .792 | .010 | -0.465 | .107 | .097 | -0.255 | .376 | .032 |
|  | exponent | -0.093 | .326 | .049 | -0.027 | .780 | .000 | 0.037 | .781 | .003 | 0.053 | .692 | .006 |
|  | offset | -0.035 | .693 | .098 | -0.025 | .779 | .009 | -0.139 | .269 | .048 | -0.011 | .934 | .000 |
| fronto-central | theta | -0.044 | .678 | .203 | -0.080 | .453 | .038 | -0.331 | **.029*** | .166 | -0.005 | .972 | .000 |
|  | alpha | -0.094 | .437 | .258 | -0.145 | .230 | .118 | -0.354 | **.038*** | .152 | -0.047 | .781 | .003 |
|  | beta | -0.072 | .462 | .134 | -0.145 | .141 | .083 | -0.136 | .329 | .038 | 0.075 | .591 | .012 |
|  | gamma | -0.007 | .962 | .006 | -0.089 | .539 | .012 | 0.094 | .647 | .009 | 0.068 | .740 | .005 |
|  | exponent | 0.020 | .771 | .010 | -0.004 | .955 | .001 | -0.089 | .363 | .033 | 0.020 | .836 | .002 |
|  | offset | 0.024 | .739 | .015 | -0.048 | .505 | .006 | -0.112 | .266 | .049 | 0.053 | .598 | .011 |
| centro-parietal | theta | -0.020 | .859 | .121 | -0.107 | .343 | .021 | -0.283 | .076 | .116 | 0.087 | .584 | .012 |
|  | alpha | -0.068 | .632 | .216 | -0.137 | .334 | .086 | -0.435 | **.030*** | .164 | -0.060 | .766 | .004 |
|  | beta | 0.004 | .948 | .128 | -0.130 | **.038*** | .154 | -0.203 | **.022*** | .178 | 0.043 | .626 | .010 |
|  | gamma | -0.032 | .639 | .050 | -0.118 | .087 | .148 | -0.046 | .634 | .009 | 0.035 | .723 | .005 |
|  | exponent | 0.037 | .399 | .001 | -0.006 | .896 | .007 | -0.066 | .288 | .045 | 0.037 | .544 | .015 |
|  | offset | 0.047 | .433 | .009 | -0.054 | .371 | .008 | -0.138 | .105 | .099 | 0.065 | .443 | .024 |
| occiptal | theta | -0.051 | .845 | .097 | 0.179 | .496 | .008 | -0.528 | .155 | .078 | -0.531 | .152 | .079 |
|  | alpha | -0.192 | .440 | .183 | 0.070 | .777 | .020 | -0.463 | .187 | .068 | -0.396 | .260 | .050 |
|  | beta | -0.087 | .675 | .074 | 0.062 | .766 | .003 | -0.239 | .414 | .027 | -0.199 | .497 | .019 |
|  | gamma | -0.080 | .733 | .006 | 0.109 | .645 | .000 | 0.036 | .915 | .000 | -0.185 | .579 | .013 |
|  | exponent | -0.005 | .944 | .069 | 0.019 | .790 | .025 | -0.133 | .195 | .065 | -0.123 | .230 | .056 |
|  | offset | -0.041 | .759 | .088 | 0.078 | .555 | .018 | -0.220 | .242 | .054 | -0.289 | .125 | .090 |
| global | theta | -0.037 | .727 | .225 | -0.057 | .594 | .032 | -0.386 | **.011*** | .212 | -0.042 | .781 | .003 |
|  | alpha | -0.105 | .411 | .288 | -0.107 | .402 | .095 | -0.418 | **.021*** | .182 | -0.106 | .558 | .014 |
|  | beta | 0.001 | .988 | .220 | -0.078 | .248 | .131 | -0.304 | **.002**** | .296 | -0.065 | .501 | .019 |
|  | gamma | 0.104 | .381 | .000 | 0.015 | .899 | .017 | -0.215 | .201 | .064 | -0.142 | .399 | .029 |
|  | exponent | -0.044 | .465 | .045 | -0.051 | .406 | .003 | -0.005 | .954 | .000 | 0.076 | .373 | .032 |
|  | offset | -0.024 | .721 | .102 | -0.064 | .337 | .032 | -0.117 | .212 | .061 | 0.039 | .677 | .007 |

*Individual contrast p-values are reported uncorrected, as they decompose the FDR-corrected omnibus tests reported in Supplementary Table S5. ROI = region of interest; β = unstandardized regression coefficient (log-ratio units for frequency band power; raw units for aperiodic parameters); sp-R² = semi-partial R². β (EC vs. EO) and β (Narrow vs. EO) = individual condition contrasts against Stance-EO. Significant values are shown in bold with * p < .05, ** p < .01, and *** p < .001*
